# Local retention efficacy of steroid-loaded PLGA microspheres in epidural injection

**DOI:** 10.1038/s41598-022-16359-0

**Published:** 2022-07-18

**Authors:** Sowon Jang, Jungheum Cho, Eugene Lee, Yusuhn Kang, Myung Joo Kang, Young Wook Choi, Joon Woo Lee

**Affiliations:** 1 Department of Radiology, Seoul National University Bundang Hospital, Seoul National University College of Medicine, 82, Gumi-ro 173 Beon-gil, Bundang-gu, Seongnam-si, Gyeonggi-do 13620 Republic of Korea; 2 College of Pharmacy, Dankook University, 119, Dandae-ro, Dongnam-gu, Cheonan-si, Chungnam 31116 Republic of Korea; 3 College of Pharmacy, Chung-Ang University, 84, Heukseok-ro, Dongjak-gu, Seoul, 06974 Republic of Korea

**Keywords:** Medical research, Experimental models of disease

## Abstract

Long-term effects of epidural steroid injections for pain management require novel drug formulations that increase tissue retention time. Present study aimed to investigate the local retention of steroid-loaded poly(lactic-co-glycolic acid) (PLGA) microspheres in epidural injection using a rabbit model. Twenty rabbits were randomly assigned to a PLGA group (n = 10) and a triamcinolone acetonide (TA) group (n = 10). Each animal was injected with either TA-loaded PLGA microspheres or conventional TA suspension into the lumbar epidural space. The lumbar segments were then harvested from the sacrificed rabbits on day 1, week 1, 2, and 4 after the injection. On day 1, the residual steroid concentration (RSC) was lower in the PLGA group than in the TA group (5.03 ppm vs. 13.01 ppm). However, after a week, more steroids remained in the PLGA group (3.29 ppm vs. 0.58 ppm). After 2 weeks, fewer steroids remained in the PLGA group than in the TA group, although both contained less than 10% of the initial retention dose. This study shows that steroid-loaded PLGA tended to have higher steroid retention in tissue than the steroid itself at the first week after epidural injection. However, most of the steroids disappeared after 2 weeks in both groups.

## Introduction

Since its introduction in 1953^1,2^, epidural steroid injection (ESI) has been widely used in patients with chronic back pain and plays a pivotal role in pain management. Chronic back pain is defined as pain lasting for more than 6 months and is not well controlled by routine pain management^3,4^. ESI is a procedure that injects glucocorticoids and anesthetics and is thought to relieve pain by reducing nerve-root inflammation and ischemia^5^. A major disadvantage of ESI is its short-lasting effect (2 weeks to 6 months) in pain management^6–8^. There are various ESI recommendations based on clinical guidelines^8–10^. But most guidelines limit the number of ESIs to three in 6 months and six in 1 year^3,6^ due to concerns about adverse effects such as steroid side effects^11–14^, injection-related complications, and radiation exposure. Therefore, frequently relapsed back pain is difficult to manage with current ESI regimen.

In this respect, there is a need for a formulation lasting longer than conventional drugs after a single injection. The size of the formulation should be small enough not to cause embolic infarction. Recently, pharmaceutical approaches using hydrogels, liposomes, nanoparticles, and microparticles have allowed a prolonged retention time and slow dissolution of the drug^15–21^. Poly(lactic-co-glycolic acid) (PLGA), approved by US Food and Drug Administration, is the most popular synthetic biodegradable polymer widely used for drug encapsulation^22–24^. PLGA has advantages in feasibility to design sustained releases, biodegradability, and biocompatibility. PLGA easily degraded to carbon dioxide and water in the body via Krebs cycle^23,25,26^. Desired drug release could be effectively achieved by adjusting factors affecting the biodegradation of PLGA, including material, processing, and physiological factors^23^. Previous studies reported improvement in local steroid retention in the knee joints and paraspinal muscles of rats using PLGA microsphere^27–30^. Therefore, we hypothesized that a steroid-loaded PLGA microsphere as an ESI could lead to an important breakthrough in controlling chronic back pain by increasing the duration of the drug effects and by avoiding the side-effects from repeated injections. To the best of our knowledge, no preclinical in vivo study has directly applied the PLGA formulation as an ESI. This study aimed to compare the local retention of steroid-loaded PLGA microspheres with that of the steroid itself after epidural injection through an in vivo study using a rabbit model.

## Materials and methods

The study protocols were authorized and approved by the Animal Care and Use Committee of our Institute (approval number: BA1608-206/050-01) before the commencement of the study and reported in accordance with ARRIVE guidelines. All animal experiments were performed in compliance with the Principles of Laboratory Animal Care (NIH publication number 85-23, revised 1996) and the approved guidelines. This experimental study was conducted from July 2018 to August 2019.

### Animals

Twenty-five female New Zealand White rabbits weighing 3.6 ± 0.4 kg (range, 3.0–4.0 kg) were obtained from DooYeol Biotech (Seoul, Korea), and Saeronbio (Uiwang-si, Korea). The rabbits were acclimatized to the animal experimental platform for 1 week prior to the experiments. The animals were housed in individual cages under standard laboratory conditions including a controlled light cycle (12-h light/12-h dark) and controlled temperature (21 ± 2 ºC). Tap water and standard laboratory chow were provided ad libitum.

**Figure 1:**
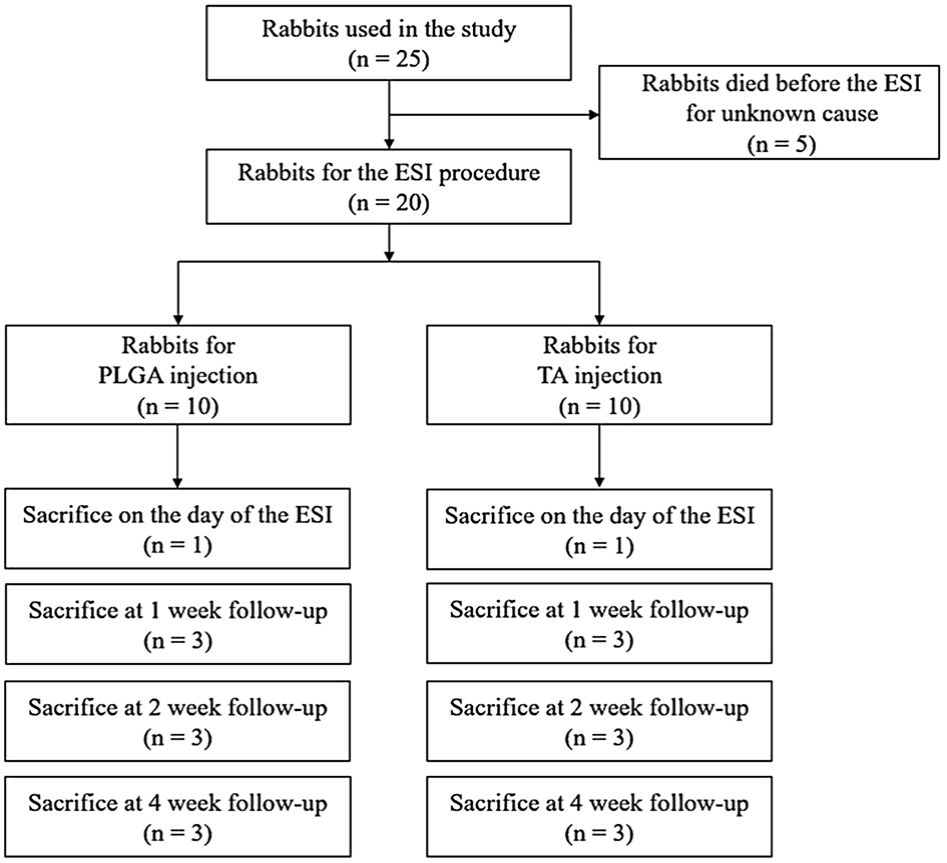
Schematic diagram of the study design. Flow diagram showing the study design.

We initially planned to use 20 rabbits. However, a total of 25 rabbits were used in the experiment, as 5 of them died for unknown reasons immediately after anesthesia or during transport (Fig. 1). Rabbits were randomly assigned to an experimental group (n = 10, PLGA group) or a control group (n = 10, triamcinolone acetonide (TA) group). To assess the local steroid retention in the lumbar segments over time, we sacrificed the rabbits in both groups at scheduled time intervals from the day of the epidural injection, namely, on day 1 (n = 1 for each group), and at weeks 1, 2, and 4 (n = 3 for each group and each time point).

### Preparation of the PLGA microspheres

The TA-loaded polymeric microspheres were prepared using a Buchi mini-spray dryer (Model B-290, Buchi Labortechnik AG, Flawil, Switzerland). TA powder (2000 mg), PLGA polymer (4000 mg, lactide/glycolide ratio of 50:50, molecular weight 38,000–54,000 Da, 5050 DLG 4A, Lakeshore Biomaterials, Birmingham, AL, USA), and lecithin (200 mg, Avanti Polar Liqids, Alabaster, AL, USA) were completely dissolved in dichloromethane (260 ml, 99.9% HPLC grade, Dichloromethane, Daejung, Seoul, South Korea) at 250 rpm for 2 h using a magnetic stirrer. The organic solution was then drained into the spray dryer at a feeding rate of 5 ml/min. The organic solvent was evaporated with inlet and outlet temperatures of 40 °C and 28 °C, respectively. The aspirator capacity was set to 100%. The collected TA-loaded PLGA microspheres were stored in a desiccator at room temperature. For epidural injection, the spray-dried particles were re-dispersed in an aqueous medium containing polysorbate 80 (0.04 v/v%, TWEEN 80, Sigma-Aldrich, St. Louis, MO, USA), sodium carboxymethylcellulose (0.63 w/v%, Sigma-Aldrich, St. Louis, MO, USA), and sodium chloride (0.66 w/v%, Sigma-Aldrich, St. Louis, MO, USA), as the dispersant, suspending agent, and isotonic agent, respectively.

**Figure 2:**
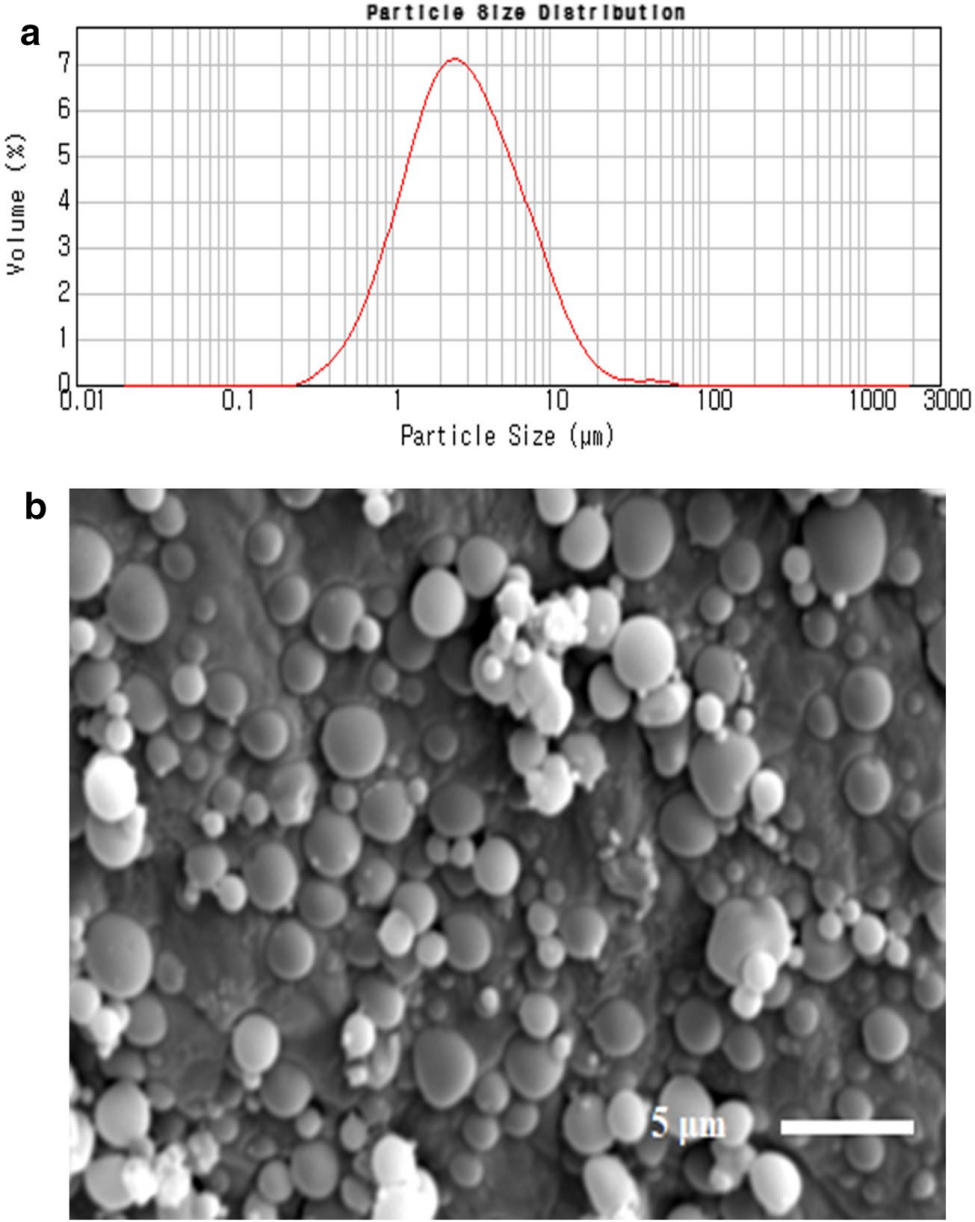
Morphologic and physical characteristics of TA-loaded PLGA microspheres. (**a**) Particle size distribution of TA-loaded PLGA microspheres prepared using spray drying technique. (**b**) Electron microscopy image of PLGA microspheres.

The particle size and homogeneity of the TA-loaded PLGA microspheres were determined using a laser diffraction particle size analyzer (LA-950, Horiba Ltd., Kyoto, Japan). The TA-loaded PLGA microspheres fabricated by the spray-drying procedure were highly spherical, with a smooth and homogeneous surface (Fig. 2). The loading efficiency of TA microcrystals on PLGA microsphere was greater than 95%.

### Experimental design and epidural injection

**Figure 3:**
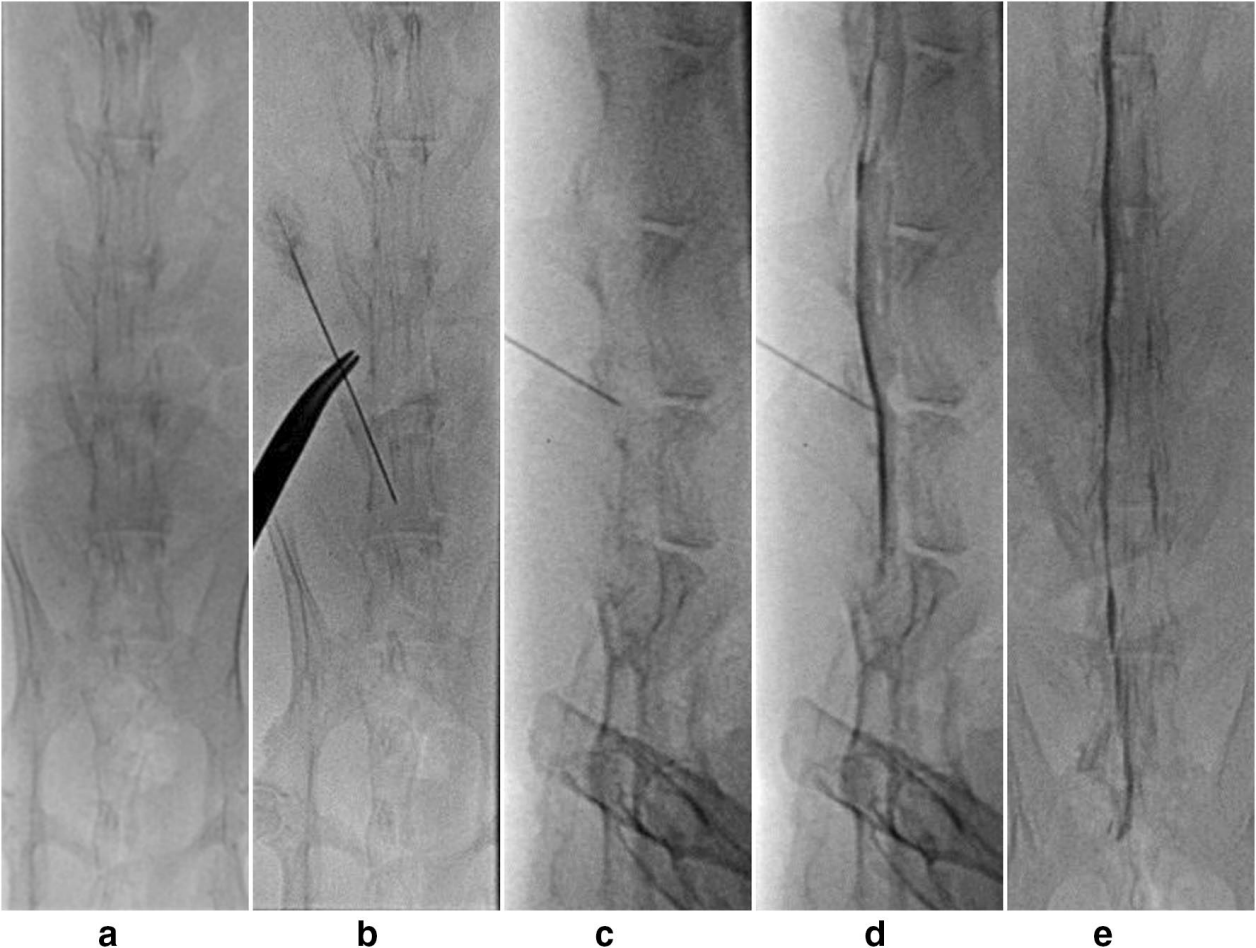
Epidural steroid injection via the interlaminar route. (**a**) Adequately anesthetized rabbit is placed on a fluoroscopy procedure table in the prone position. (**b**,**c**) Epidural injection is administered at the L6/L7 vertebral segment under fluoroscopic guidance. The targeted level is confirmed on both lateral (**d**) and anteroposterior (**e**) views.

All experiments were conducted in fully anesthetized animals. Anesthesia was performed via intramuscular injection of alfaxalone (Alfaxan, 10 mg/ml; Jurox Pty, Ltd., Rutherford, Australia; 5 mg/kg body weight) and xylazine (Rompun, 23.32 mg/ml; Bayer Korea, Ansan, Korea; 5 mg/kg body weight) for all 20 rabbits before ESI. The anesthetized rabbit was placed on a fluoroscopy table in the prone position. The target level, the interspinous space between the L6 and L7 vertebrae, was identified by both manual palpation and fluoroscopic guidance. Epidural puncture was performed using a 25-gauge spinal Quincke needle after sterilization. The correct positioning of the needle tip in the epidural space was confirmed by injection of 0.5 ml of contrast material (iohexol, Omnipaque 300, 300 mg iodine per ml; GE Healthcare Co., Ltd., Shanghai, China) under fluoroscopy (Fig. 3). In the experimental group, 1.5 ml of PLGA microspheres (containing 40 mg of TA suspended in 1.5 ml) was manually injected via the extension line. The rabbits in the control group received 1.5 ml TA suspension (1 ml TA [Triam, 40 mg/ml; Shinpoong Pharmaceuticals, Seoul, Korea] and 0.5 ml normal saline) in the same way. After injection of each formulation, 0.5 ml contrast medium was infused through the extension line to inject the drug remaining in the line. All epidural injections were performed by a single radiologist (S.J, 3 years of experience) under the supervision of an experienced radiologist (J.W.L, 20 years of experience). After the initial injection, animals were housed in an animal experimental platform and cared for by the veterinarians.

### Euthanization and tissue harvesting

**Figure 4:**
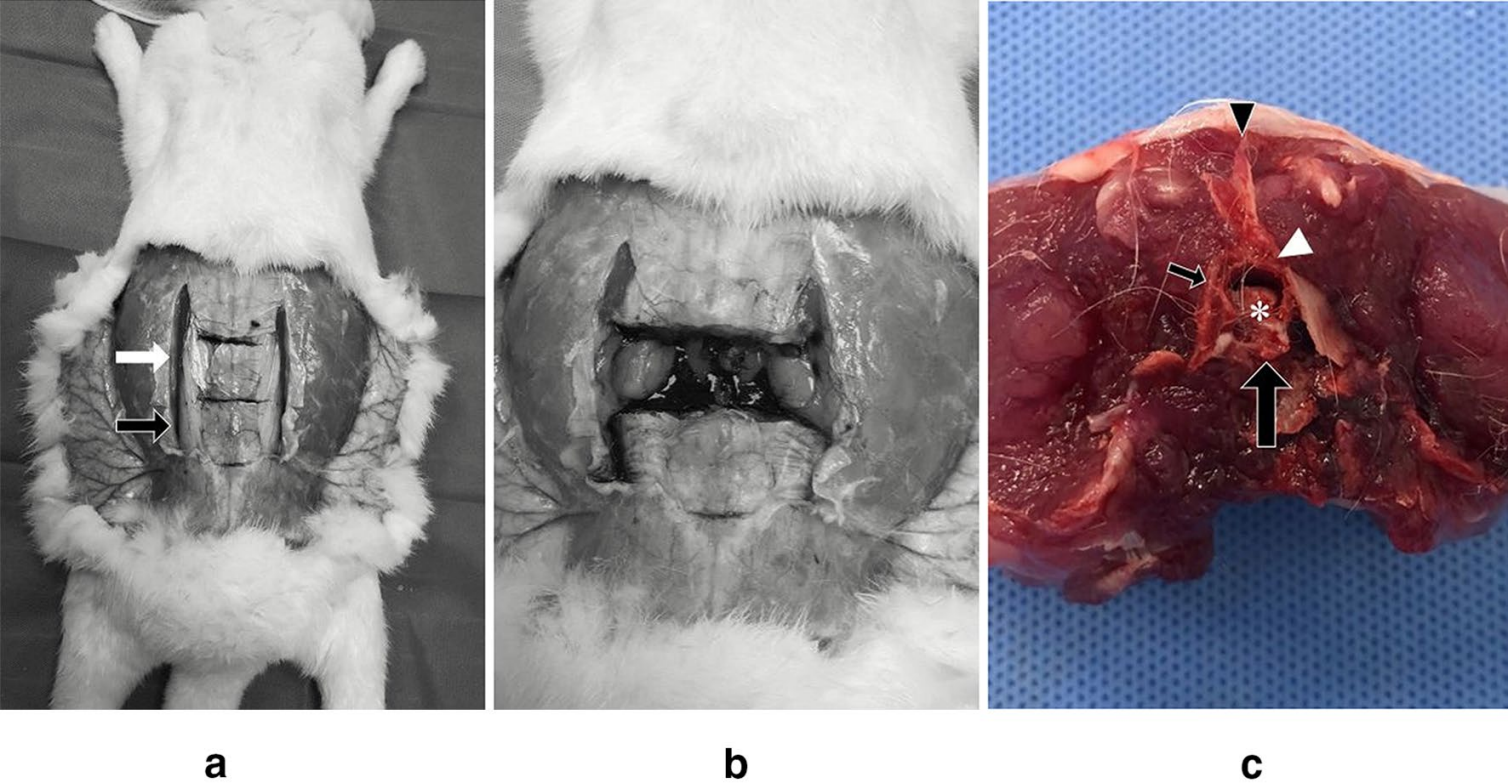
Tissue harvesting. (**a**) The euthanized rabbit is placed in prone position. Two consecutive vertebrae consisting of the ESI level (black arrow) and the level just above (white arrow) are harvested simultaneously. (**b**) A single vertebral segment was harvested. (**c**) Axial view of the harvested vertebral segment shows potential epidural space (white arrowhead) just beneath the bony neural arch (small black arrow). The neural spine (black arrowhead) of rabbit vertebra was located on the dorsal side, and the vertebral body (large black arrow) is located on the ventral side. The spinal cord (asterisk) was visualized within the neural canal.

The tissue was harvested from the sacrificed animals at the predetermined time intervals after the epidural injection. In each group, the rabbits were anesthetized by performing consecutive intramuscular and intravenous injection of alfaxalone (Alfaxan, 10 mg/ml; Jurox Pty, Ltd., Rutherford, Australia) and xylazine (Rompun, 23.32 mg/ml; Bayer Korea, Ansan, Korea) as follows: intramuscular injection with alfaxalone (0.7 ml/kg) and xylazine (0.3 ml/kg) followed by intravenous injection with alfaxalone (0.35 ml/kg) and xylazine (0.15 ml/kg). The fully anesthetized rabbits were euthanized by intravenous injection of potassium chloride (150 mg/ml; JW Pharmaceutical, Seoul, Korea; 150 mg/kg body weight). After death was confirmed by loss of heart rate, two consecutive lumbar segments comprising the injection segment (L6/L7) and the adjacent segment (L4/L5) were harvested (Fig. 4). All tissue samples were kept separately on ice until further chemical analysis was performed. All instruments used for dissection were rinsed thoroughly before processing the next sample.

### Tissue processing and quantitation of triamcinolone acetonide

The residual steroid concentration (RSC) in the lumbar segments was analyzed using a high-performance liquid chromatography-mass spectrometry/mass spectrometry (HPLC–MS/MS) assay in a chemistry laboratory (Biological Mass Spectrometry Group at the Dankook University, Cheonan, Korea). First, the tissue was processed by adding 50 ml methanol to each bottle containing the tissue and agitating for 24 h in a shaking incubator. Ten microliters of the supernatant obtained by centrifugation of the diluted solution at 14,000 rpm for 10 min was analyzed by an HPLC–MS/MS system (LC-20 Prominence HPLC system; Shimadzu, Tokyo, Japan) and an API 2000 triple quadrupole mass spectrometer (AB/SCIEX, Poster City, CA). Chromatographic separation was performed using a Phenomenex Luna C18 column (2.0 × 150 mm, 5-µm particle size). The mobile phase had a flow rate of 0.25 ml/min and was composed of 0.1% formic acid, with the same volume ratio of water and acetonitrile. The electrospray ionization technique with positive ion mode was used to analyze the steroidal compound eluted from the column, after being transferred into the MS/MS instrument, under the following conditions: gas temperature, 400℃; ion spray voltage, 5500 V; curtain gas pressure, 16 psi; and collision gas pressure, 6 psi. TA was monitored and confirmed based on the transitions (m/z 435.1 to 415.0 transition for monitoring; 435.1 to 171.4 and 435.1 to 397.1 for confirmation). The assay exhibited excellent linearity (R^2^ value of 0.9973) over the TA concentration range. Each sample was analyzed three times in the same way.

### Statistical analysis

Distribution normality of the data was evaluated using the Spiro-Wilk normality test. Data with normal distribution were expressed as mean and standard deviation (SD), while non-normally distributed data were presented as median and interquartile range (IQR). Experimental data were presented descriptively as percentages and plot charts. Generalized estimating equations were applied to assess the difference in the RSC between the drugs over time. Analyses were done with R software (version 4.0.2; The R Project for Statistical Computing), and *P*-values less than 0.05 were considered statistically significant.

## Results

**Figure 5:**
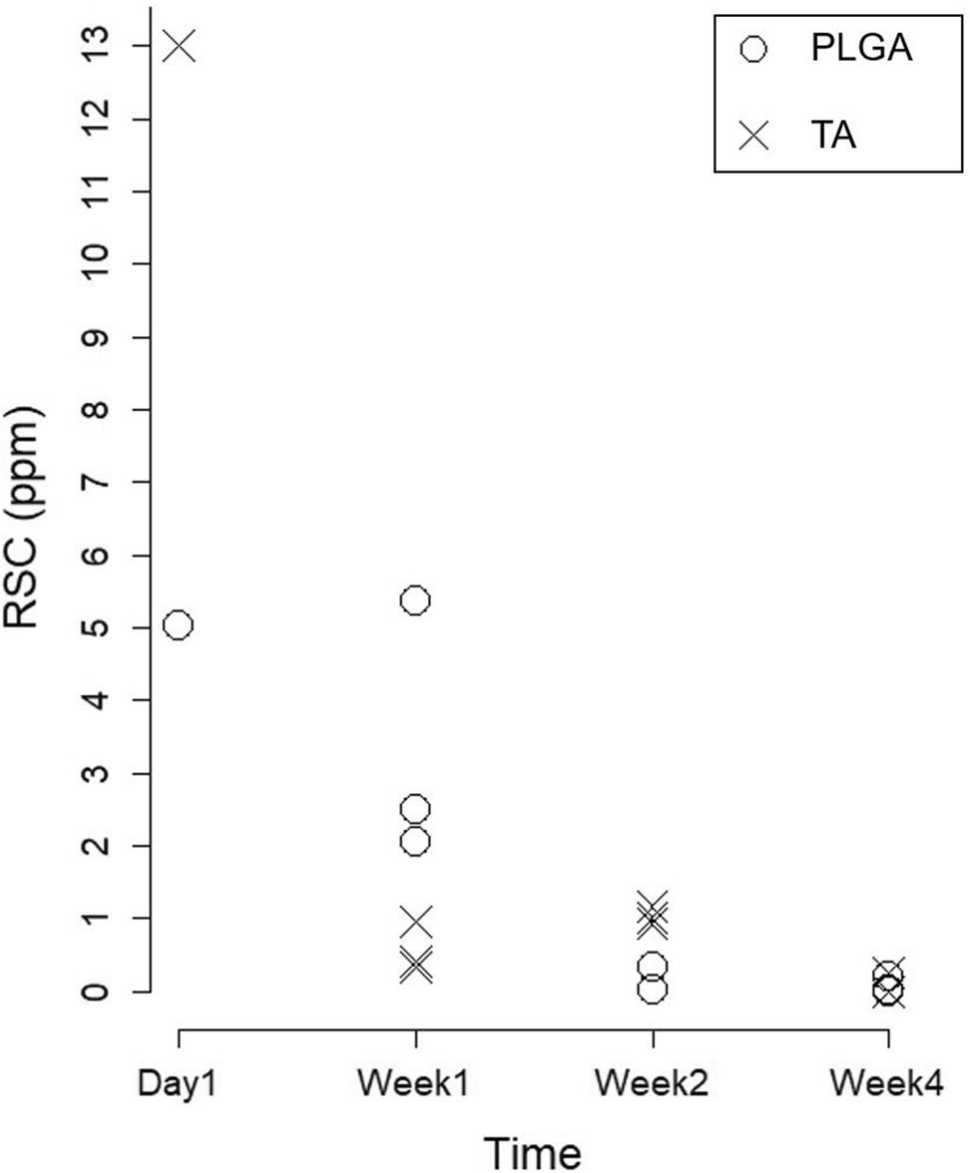
The RSC values of the injection segment in each time points.

The morphological and physical characteristics of the TA-loaded PLGA microsphere are shown in Fig. 2. The median diameter (d50) of the TA-loaded PLGA microspheres was determined to be 2.77 μm, with a narrow size distribution (span value 2.71). The mean weight of the extracted lumbar segments was 50.0 ± 4.5 mg. The RSC values of the animals are shown in Table 1 and Fig. 5. To intuitively compare RSCs, the values were expressed as a percentage of each group’s day 1 results (Table 1).

**Table 1:** Residual steroid concentration (RSC) in the extracted spinal segments according to the time-points, drug formulations, and locations.

	PLGA		TA	
	Injection segment (ppm)	Adjacent segment (ppm)	Injection segment (ppm)	Adjacent segment (ppm)
**Day 1**				
A	100% (5.03 ± 0.16)	100% (1.58 ± 0.14)	100% (13.01 ± 0.64)	100% (4.95 ± 0.13)
**Week 1**				
A	106.6% (5.36 ± 0.08)	10.1% (0.16 ± 0.02)	3.2% (0.41 ± 0.03)	2.4% (0.12 ± 0.01)
B	49.5% (2.49 ± 0.06)	10.8% (0.17 ± 0)	7.5% (0.97 ± 0.08)	7.1% (0.35 ± 0.02)
C	40.6% (2.04 ± 0.01)	82.9% (1.31 ± 0.03)	2.7% (0.35 ± 0.02)	3.6% (0.18 ± 0.01)
**Week 2**				
A	0.2% (0.01 ± 0.01)	3.8% (0.06 ± 0)	7.2% (0.94 ± 0.01)	7.9% (0.39 ± 0.01)
B	6.4% (0.32 ± 0.01)	2.5% (0.04 ± 0)	9.1% (1.18 ± 0.04)	8.5% (0.42 ± 0.03)
C	0.2% (0.01 ± 0.01)	8.9% (0.14 ± 0.01)	7.8% (1.01 ± 0.02)	9.1% (0.45 ± 0.03)
**Week 4**				
A	4.0% (0.20 ± 0.02)	Not detected	0.1% (0.01 ± 0)	0.2% (0.01 ± 0)
B	Not detected	Not detected	2.0% (0.26 ± 0.01)	2.8% (0.14 ± 0.01)
C	0.4% (0.02 ± 0.01)	Not detected	0.1% (0.01 ± 0)	Not detected

Table 1 presents the raw data of the study results. Values are expressed as mean ± standard deviation, because each sample was analyzed three times for accuracy. To intuitively compare RSCs, the values obtained from animals in each group sacrificed immediately after ESI (Day 1) were used as standard (set 100%, relatively).

*RSC* residual steroid concentration, *PLGA* poly (lactic-co-glycolic acid), *TA* triamcinolone acetonide, *ESI* epidural steroid injection.

Regarding the injected segment, the RSC values obtained immediately after ESI (day 1) were lower in the PLGA group than in the TA group (5.03 ± 0.16 ppm vs. 13.01 ± 0.64 ppm). In the PLGA group, the steroid remained almost completely in an animal at week 1, while about half of the steroids were detected in the other two animals at week 1 (5.36 ± 0.08, 2.49 ± 0.06, and 2.04 ± 0.01 ppm). However, after 2 weeks from the injection, the RSC values were less than 10% of the initial retention (0.01 ± 0.01, 0.32 ± 0.01, and 0.01 ± 0.01 ppm). In the TA group, all week 1 values (0.41 ± 0.03, 0.97 ± 0.08, and 0.35 ± 0.02 ppm) and week 2 values (0.94 ± 0.01, 1.18 ± 0.04, and 1.01 ± 0.02 ppm) were less than 10% of its day 1 RSC value. At week 4, extremely small amount of RSCs were detected in both groups, which were less than 5% of the initial RSC values.

**Figure 6:**
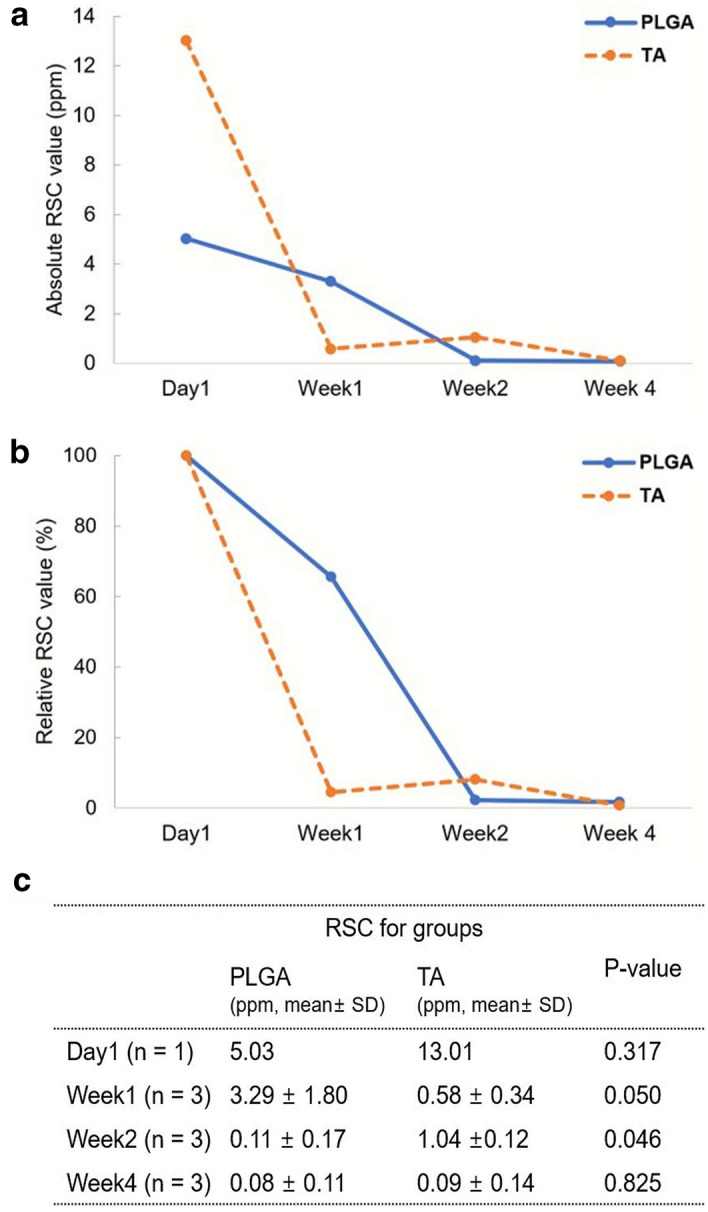
Trend of RSC over time in the injection segment of each group. The RSC trends over time was investigated using mean ± standard deviation of each group and time-point. The RSC were presented as absolute RSC value (**a**) and relative RSC value (**b**) expressing as a percentage of the RSC recorded on day 1 of each group. Steroid-loaded PLGA showed a tendency of slow degradation during the initial period. (**c**) *P*-value obtained from generalized estimating equations employed to investigate whether the interaction between time and the formulation affects the RSC value.

When investigating the RSC trend over time, the slope of RSC reduction was milder in the PLGA group than in the TA group during the first week after injection (Fig. 6). Compared to the TA group, the PLGA group had a higher RSC at week 1 despite a low initial steroid retention (106.6%, 49.5%, and 40.6% in PLGA group; 3.2%, 7.5%, and 2.7% in TA group, Table 1). However, at week 2, the PLGA group showed fewer steroid retention than in TA group, although both contained less than 10% of the initial retention dose (0.2%, 6.4%, and 0.2% in PLGA group; 7.2%, 9.1%, and 7.8% in TA group; Table 1). The RSC values differed depending on the groups over time (*P* < 0.001). The RSC value was significantly higher in the injection segment (L6/L7) than in the adjacent segment (L4/L5) in both groups (*P* = 0.012).

No gait complications were noted in the animals before euthanization, except for a rabbit in the TA group, which was sacrificed at week 2 with spinal cord injury during the ESI procedure.

## Discussion

In this study, the injection segment showed more steroid retention than the adjacent segment in both PLGA and TA group. The amount of steroid retention in the lumbar segments decreased over time in both groups. These trends of steroid retention indicate the validity of our animal model, which was developed in previous studies^30,31^. Steroid retention showed different trends depending on the drug formulation. When using PLGA microspheres, nearly half of the steroids remained in the tissue at first week despite the lower initial retention compared to injection of the steroid itself. However, using PLGA microspheres were associated with less steroid retention after 2 weeks compared to using steroid itself.

Week 1 results of this study are consistent with published studies. Previous studies have reported that small delivery systems allow for slow dissolution of drugs and steady drug-tissue binding in both in vivo and in vitro environments^16–21,32^. In particular, the PLGA microspheres showed increased drug retention in the knee and the paraspinal muscles after intra-articular and intramuscular injection^27–30^. However, in this study, the PLGA microspheres improved steroid retention within the epidural space only during the first week after ESI.

In current study, the use of PLGA microspheres reduced steroid retention at week 2. However, too few steroids remained in both groups to expect any clinical effect. This unexpected result is probably due to leakage from the epidural space, phagocytosis, and biodegradation of the microspheres. The epidural space is anatomically and compositionally different from paraspinal muscle or cartilage of the knee joint used in previous studies. The epidural space is not a confined structure so that injected drugs can spread immediately into the epidural space and can be leaked through neural foramina. In addition, the epidural space is a fat-containing structure rich in lymph and vascular channels that transport macrophages. The injected drug is removed from the body through the phagocytosis of macrophages—the smaller the target particle size, the more active the reaction^33–36^. Moreover, the maximal phagocytosis occurred in particles with 2–3 um which was used in current study to prevent serious leaks expected when using smaller particles and to avoid the embolic infarction expected when using larger particles^35–37^. The in vivo biodegradation of PLGA is influenced by tunable factors including physical and biochemical properties of the material such as size, molecular weight, and surface charge. In particular, the degradation rate and release of the drug is generally accelerated as the molecular weight of PLGA decreases^38^. Therefore, in this study, relatively low molecular weight of PLGA may have contributed to its rapid biodegradation, fast drug release, and short-term drug retention in tissues.

Nevertheless, the fact that the PLGA reduced steroid retention more slowly during the first week suggests potential for clinical use. In pain management with ESI, it is important to effectively reduce inflammation at an early stage. In the same context, keeping the drug concentration constant above the threshold is important to achieve therapeutic effect. The PLGA has an advantage of allowing higher drug retention at an early phase without sudden dose peaks in vivo with injection. Another benefit may be that the PLGA induces low steroid retention on the day of injection. This is because sudden dose peaks can be associated with systemic side effects of single steroid injections. In addition, the PLGA showed significantly lower plasma level than conventional drugs when the same amount was administered^27^. Therefore, PLGA has the potential to contribute to ESI once an appropriate injection dose is established. Future injection dose should be established to maintain the maximum therapeutic dose in the tissue without increasing plasma concentrations. Further studies are needed to determine the injection dose or to optimize the properties of the PLGA formulation—such as particle size, hydrophilic properties, ionic character—for local retention.

Some limitations of our experimental study must be acknowledged. First, it may be difficult to generalize our results because we used a small number of rabbits in an experimental design based on the 3R principle (replace, reduce, refine)—designed to generate the maximum amount of knowledge using the smallest possible number of animals. Another limitation is that the incidence of embolic infarction was not monitored except prior to sacrifice, as the experiment focused on determining the topical maintenance efficacy of the new formulation. Further studies would be needed to evaluate the safety and complications associated with the formulation. Third, we used a single size of microspheres and performed only the midline approach to the lumbar spine in all animals. Experimentation with different sized particles or other injection methods such as facet joint or transforaminal approach may give different results. Fourth, histopathologic analysis was not conducted in this study. This is because the focus of the study was to compare the quantity of remaining microspheres depending on the drug formulation. Finally, researchers have tried to inject the drug identically, but the distribution of the drug may vary from individual to individual. For example, in some animals, the injected drug may have been lost immediately through the neural foramen of the spine.

In summary, more steroids remained in the epidural space during the first week after PLGA injection despite the low initial retention compared to conventional drugs. Our findings suggest the potential for PLGA use in ESI if injection doses are established and formulation properties are adjusted. Further research is needed to clarify this point.

## Data Availability

Data are available on reasonable request.

## References

[1] Lievre JA, Bloch-Michel H, Attali P (1957). Trans-sacral injection; clinical and radiological study. Bull. Mem. Soc. Med. Hop Paris.

[2] Lievre JA (1953). L’hydrocortisone en injection locale. Rev. Rhum. Mal. Osteoartic..

[3] Boswell MV (2007). Interventional techniques: Evidence-based practice guidelines in the management of chronic spinal pain. Pain Physician.

[4] Hartvigsen J (2018). What low back pain is and why we need to pay attention. Lancet.

[5] Harrast MA (2008). Epidural steroid injections for lumbar spinal stenosis. Curr. Rev. Musculoskelet. Med..

[6] Kreiner DS, Shaffer WO, Toton J, Baisden J, Gilbert T, Evidence-based Clinical Guidelines Committee (2011). Evidence-Based Clinical Guidelines for Multidisciplinary Spine Care: Diagnosis and Treatment of Degenerative Lumbar Spinal Stenosis.

[7] Chou R (2015). Epidural corticosteroid injections for radiculopathy and spinal stenosis: A systematic review and meta-analysis. Ann. Intern. Med..

[8] Stochkendahl MJ (2018). National Clinical Guidelines for non-surgical treatment of patients with recent onset low back pain or lumbar radiculopathy. Eur. Spine J..

[9] Qaseem A, Wilt TJ, McLean RM, Forciea MA (2017). Noninvasive treatments for acute, subacute, and chronic low back pain: A clinical practice guideline from the American College of Physicians. Ann. Intern. Med..

[10] UK National Institute for Health and Care Excellence*. Low Back Pain and Sciatica in Over 16s: Assessment and Management*. 33090750

[11] Botwin KP (2000). Complications of fluoroscopically guided transforaminal lumbar epidural injections. Arch. Phys. Med. Rehabil..

[12] Huston CW, Slipman CW, Garvin C (2005). Complications and side effects of cervical and lumbosacral selective nerve root injections. Arch. Phys. Med. Rehabil..

[13] McGrath JM, Schaefer MP, Malkamaki DM (2011). Incidence and characteristics of complications from epidural steroid injections. Pain Med..

[14] Engel A, King W, MacVicar J (2014). The effectiveness and risks of fluoroscopically guided cervical transforaminal injections of steroids: A systematic review with comprehensive analysis of the published data. Pain Med..

[15] Jusu SM (2020). Drug-encapsulated blend of PLGA-PEG microspheres: In vitro and in vivo study of the effects of localized/targeted drug delivery on the treatment of triple-negative breast cancer. Sci. Rep..

[16] Ho MJ, Kim SR, Choi YW (2019). Recent advances in intra-articular drug delivery systems to extend drug retention in joint. J. Pharm. Investig..

[17] Rudnik-Jansen I (2019). Applicability of a modified rat model of acute arthritis for long-term testing of drug delivery systems. Pharmaceutics.

[18] Luzardo-Alvarez A, Lamela-Gomez I, Otero-Espinar F, Blanco-Mendez J (2019). Development, characterization, and in vitro evaluation of resveratrol-loaded poly-(epsilon-caprolactone) microcapsules prepared by ultrasonic atomization for intra-articular administration. Pharmaceutics.

[19] He Z, Wang B, Hu C, Zhao J (2017). An overview of hydrogel-based intra-articular drug delivery for the treatment of osteoarthritis. Colloids Surf. B.

[20] Rudnik-Jansen I (2017). Prolonged inhibition of inflammation in osteoarthritis by triamcinolone acetonide released from a polyester amide microsphere platform. J. Control Release.

[21] Zhang Z (2016). Intra-articular injection of cross-linked hyaluronic acid-dexamethasone hydrogel attenuates osteoarthritis: An experimental study in a rat model of osteoarthritis. Int. J. Mol. Sci..

[22] Kumari A, Yadav SK, Yadav SC (2010). Biodegradable polymeric nanoparticles based drug delivery systems. Colloids Surf. B.

[23] Mir M, Ahmed N, Rehman AU (2017). Recent applications of PLGA based nanostructures in drug delivery. Colloids Surf. B.

[24] Zhang Z, Bi X, Li H, Huang G (2011). Enhanced targeting efficiency of PLGA microspheres loaded with Lornoxicam for intra-articular administration. Drug Deliv..

[25] Hines DJ, Kaplan DL (2013). Poly(lactic-co-glycolic) acid-controlled-release systems: Experimental and modeling insights. Crit. Rev. Ther. Drug Carrier Syst..

[26] Lagreca E (2020). Recent advances in the formulation of PLGA microparticles for controlled drug delivery. Prog. Biomater..

[27] Ho MJ (2019). Design and in vivo pharmacokinetic evaluation of triamcinolone acetonide microcrystals-loaded PLGA microsphere for increased drug retention in knees after intra-articular injection. Pharmaceutics.

[28] Ho MJ, Kim SR, Choi YW, Kang MJ (2016). A novel stable crystalline triamcinolone acetonide-loaded PLGA microsphere for prolonged release after intra-articular injection. Bull. Korean Chem. Soc..

[29] Kim SR (2015). Cationic PLGA/Eudragit RL nanoparticles for increasing retention time in synovial cavity after intra-articular injection in knee joint. Int. J. Nanomed..

[30] Kang Y (2018). Effect of Poly(lactide-co-glycolide) nanoparticles on local retention of fluorescent material: An experimental study in Mice. Korean J. Radiol..

[31] Cho J (2019). Quantitative assessment of steroid amount in the tissue after epidural steroid injection: A new rabbit model. Korean J. Pain.

[32] Elron-Gross I, Glucksam Y, Margalit R (2009). Liposomal dexamethasone-diclofenac combinations for local osteoarthritis treatment. Int. J. Pharm..

[33] Baranov MV, Kumar M, Sacanna S, Thutupalli S, van den Bogaart G (2020). Modulation of immune responses by particle size and shape. Front. Immunol..

[34] Paul D (2013). Phagocytosis dynamics depends on target shape. Biophys. J..

[35] Champion JA, Walker A, Mitragotri S (2008). Role of particle size in phagocytosis of polymeric microspheres. Pharm. Res..

[36] Hirota K (2007). Optimum conditions for efficient phagocytosis of rifampicin-loaded PLGA microspheres by alveolar macrophages. J. Control Release.

[37] Pacheco P, White D, Sulchek T (2013). Effects of microparticle size and Fc density on macrophage phagocytosis. PLoS ONE.

[38] Fredenberg S, Wahlgren M, Reslow M, Axelsson A (2011). The mechanisms of drug release in poly(lactic-co-glycolic acid)-based drug delivery systems: A review. Int. J. Pharm..

